# *Mycobacterium tuberculosis* PPE32 promotes cytokines production and host cell apoptosis through caspase cascade accompanying with enhanced ER stress response

**DOI:** 10.18632/oncotarget.12030

**Published:** 2016-09-15

**Authors:** Wanyan Deng, Wenmin Yang, Jie Zeng, Abualgasim Elgaili Abdalla, Jianping Xie

**Affiliations:** ^1^ State Key Laboratory Breeding Base of Eco-Environment and Bio-Resource of the Three Gorges Area, Key Laboratory of Eco-Environments in Three Gorges Reservoir Region, Ministry of Education, School of Life Sciences, Institute of Modern Biopharmaceuticals, Southwest University, Chongqing, PR China; ^2^ Key Laboratory of Molecular Biology for Infectious Diseases (Ministry of Education), Institute for Viral Hepatitis, Department of Infectious Diseases, The Second Affiliated Hospital, Chongqing Medical University, Chongqing, PR China; ^3^ Department of Clinical Microbiology, College of Medical Laboratory Sciences, Omdurman Islamic University, Omdurman, Khartoum, Sudan

**Keywords:** Mycobacterium tuberculosis, PE/PPE, macrophage, apoptosis, caspase

## Abstract

Tuberculosis, caused by *Mycobacterium tuberculosis* (MTB) infection, remains a grave global public health burden which claims the lives around two to three million annually. PE and PPE proteins, featured by the Pro-Glu (PE) or Pro-Pro-Glu (PPE) motifs at the conserved N-terminal domain, are abundant in the MTB genome. PPE32 can increase intracellular survival of mycobacteria through abnormally increase in cytokines production. PPE32 might subvert the macrophage immune response and thwart its bactericidal effect. THP-1 macrophages treated with PPE32 or infected with *Mycobacterium smegmatis* (MS) expression PPE32 showed increase of cytokines production and multiple hallmarks of apoptosis. We found that PPE32 significantly increases the expression of IL-12p40 and IL-32 through ERK1/2 signaling pathway. In addition, the cell viability of macrophage was inhibited after PPE32 stimulation. We noted that PPE32 induces cleavage of caspase-3 and caspase-9, while inhibition of caspase activity significantly abrogates the PPE32-induced cell apoptosis. Moreover, PPE32 treatment promotes endoplasmic reticulum stress related gene expression, suggesting ER stress might be responsible for PPE32-induced cell apoptosis.

## INTRODUCTION

*Mycobacterium tuberculosis* (MTB), the causative agent of human tuberculosis (TB), poses a still greater burden on human health globally (around 9.6 million people, including 5.4 million men, 3.2 million women and 1.0 million children) [1, 2]. Macrophages are the first line of defense against bacterial infection, which can secrete various cytokines to mediate the inflammatory response. During the initial stage of the disease, many mycobacterial cell wall-associated factors disturb the immune cells, including PE/PPE family protein antigens [3], crucial for the outcome of MTB infection [4]. There are 169 genes encoding PE/PPE subfamily which are named after its N terminal Pro (P)-Glu (E) and Pro (P)-Pro (P)-Glu (E) motif [5, 6]. Sixty-nine PPE proteins are classified into PPE_SVP, PPE_PPW and PPE_MPTR subfamily and 100 PE proteins belong to the PE and PE_PGRS subfamily [5, 6]. The surface location or secreted characteristics of PE/PPE proteins suggest direct interaction with host [7]. Host macrophages are usually the portal of MTB infection [8, 9]. Varied transcription level of several PE/PPE genes within macrophages or mouse during MTB infection [10] suggested roles in the interaction with host macrophage. Cell wall associated PE/PPE family proteins [11–16] might directly interact with host cell surface receptor, or even disrupts the host immunity [14, 17–20].

Many pathogenic bacteria induce host cells apoptosis by activating specific components of apoptotic pathways [21]. Macrophage apoptosis facilitates the killing of intracellular mycobacteria and triggers the adaptive immune response [22]. How mycobacteria inhibits the host cell apoptosis remains poorly understood. MTB secreted proteins can be important source of protective antigens [23]. Several secreted molecules, including PE/PPE proteins were confirmed to promote the MTB survival and persistence within the host with mechanism unknown [24–26]. MTB PE_PGRS33 activates apoptosis via activation of caspase cascade [24]. Another PE/PPE complex, PE-9 and PE10, induces macrophage apoptosis through engaging TLR4 [26]. MTB PPE32, a cell wall associated protein encoded by Rv1808, was previously found to be able to activate the cytokine profiles via TLR-2, resulting in increased bacterial burden within macrophage [14]. However, the role and mechanism of PPE32 remains elusive.

## RESULTS

### PPE32 enhances MS against stresses

**Figure 1:**
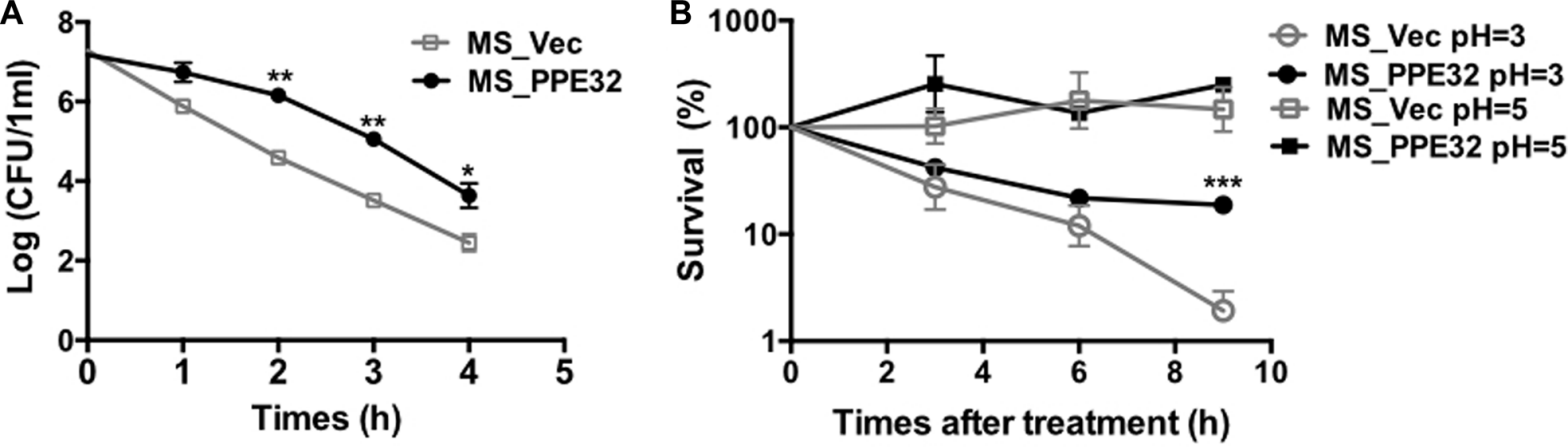
PPE32 enhanced the MS resistance to multiple extracellular stresses (**A**) Survival of MS_Vec and MS_PPE32 upon exposure to 0.05% SDS. Re-suspended 5ml recombinant MS_Vec and MS_PPE32 (*OD600* = 0.5) were exposed to 0.03% SDS for 1, 2, 3, and 4 h. And then the recombinant strains were plated onto 7H10 plates by serially ten-fold dilution (**B**) *In vitro* growth of recombinant MS_Vec and MS_PPE32 after treatment with different pH gradient for 0, 3, 6, and 9 h. The MS_Vec and MS_PPE32 strains were centrifuged, re-suspended to 5ml MB 7H9 at an OD600 of 0.5, 10-fold serial dilutions of MS_Vec and MS_PPE32 were spotted on MB 7H10 containing Kan. the bacterial numbers were counted after 3–4 days of cultivation at 37°C.

PE11 increases the permeability of mycobacterial cell wall through modifying the fatty acid components [27]. Similarly, PE19 overexpression increases membrane permeability of the MTB envelope to resist stresses encountered within the host [28]. To gain insight into whether PPE32 will affect the permeability to antimicrobial factors *in vitro*, the growth characteristics of recombinant MS_Vec and MS_PPE32 under different acid conditions and surface stresses were analyzed. As shown in the Figure 1A, although there was a rapid decrease in the bacterial numbers for all tested strains exposed to the detergent SDS (Figure 1A), MS_PPE32 was more resistant to SDS in comparison to MS_Vec, as the survival rate of MS_PPE32 was higher than MS_Vec at different intervals after SDS treatment. Acid stress mimicking the environmental cues encountered by phagocytized bacteria was set. There was no significant difference between MS_Vec and MS_PPE32 under *in vitro* acid stress at pH = 5, while the survival percentage of Ms_PPE32 was significantly higher than Ms_Vec after 9 h treatment with acid stress at pH = 3 (Figure 1B). In addition, PPE32 has no effect on the growth of MS (Figure S1). In brief, PPE32 facilitates the resistance of non-pathologic MS against surface and acid stress, as PPE32 has no effect on the *in vitro* growth of MS.

### PPE32 promotes IL-12p40 production of macrophage

**Figure 2:**
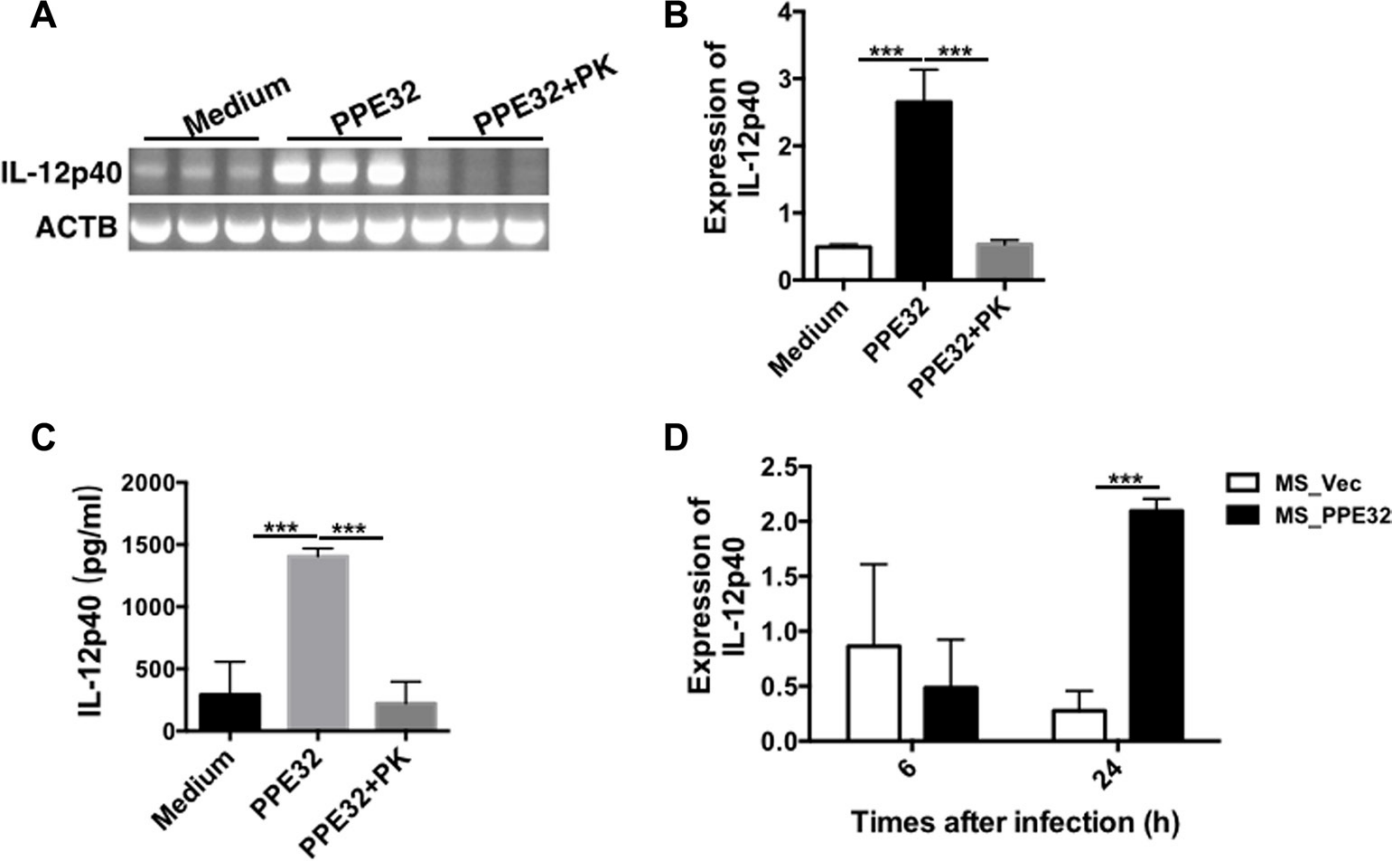
PPE32 specifically induces the expression of IL-12p40 PMA-differentiated THP-1 macrophages (2 × 10^6^/well/2 ml) were incubated with PPE32 (5 μg/ml) with or without proteinase K (PK), cells were collected and IL-12p40 mRNA levels were detected by semi-RT-PCR (**A**) and real-time RT-PCR (**B**). Culture supernatants were harvested; the secretion of IL-12p40 was detected by ELISA analysis (**C**). PMA-differentiated THP-1 macrophages were infected with MS_Vec and MS_PPE32 strains at MOI of 10 for 6 and 24 h. The IL12p40 mRNA was detected by real-time RT-PCR after MS_Vec and MS_PPE32 infection (**D**).

IL-12, originally called NK cell stimulatory factor [29] or CTL maturation factor [30], is a 70- to 75-kDa heterodimer (IL-12p70) consisting of disulfide-bonded 35-kDa (p35) and 40-kDa (p40) subunits [29, 30]. IL-12 is essential cytokine for host immunity against MTB [31–33]. IL-12 has direct stimulatory effects on effector functions of CD8^+^ T cells, which are critical for the effective control of mycobacterial infection [34, 35]. Mice deficient in endogenous IL-12 are highly susceptible to mycobacteria due to impaired type 1 cytokine responses and granuloma formation [32, 36]. IL-12p40 subunit of IL-12 has an agonistic and protective function in mycobacterial infections in mice [37]. To examine whether the PPE32-initiated signaling is critical for the generation of IL-12p40, we incubated or infected the macrophage with PPE32 protein or MS_PPE32. Accordingly, PMA-differentiated THP-1 macrophages were stimulated with 5μg/ml PPE32. The recombinant protein was found to induce IL-12p40 gene expression (Figure 2A and B) and IL-12p40 protein secretion (Figure 2C) in macrophage after 6 h incubation. Similarly, infection of MS_PPE32 resulted in an increase in IL-12p40 expression in macrophage after 24 h infection in comparison with MS_Vec (Figure 2D).

### PPE32 increases IL-32 expression

**Figure 3:**
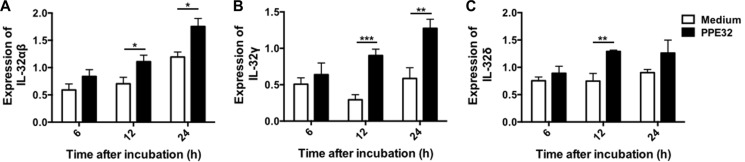
PPE32 promotes macrophage IL-32 expression Macrophages were incubated with PPE32 at the final concentration of 5 μg/ml, after 6, 12 and 24 h incubation, the expression of IL-32 isoform IL-32αβ (**A**), IL-32γ (**B**) and IL-32δ (**C**) were detected by RT-PCR.

IL-32 is a recently described cytokine produced by T lymphocytes, NK cells, epithelial cells, endothelial cells, monocytes, macrophages, T lymphocytes, eosinophils and dendritic cells [38]. IL-32 has six alternatively spliced isoforms (α, β, γ, δ, ε, and ζ) [39]. Infection of human macrophages THP-1 or PBMCs (peripheral blood mononuclear cells) with MTB H37Rv induce IL-32 expression [40, 41]. IL-32, a novel pleiotropic cytokine capable of inducing pro-inflammatory cytokines such as TNF-α and IL-1β [42] and elevated in the sera of active pulmonary TB [43], can be triggered by IL-12. We identified PPE32 has the ability to induce the expression of TNF-α, IL-6, IL-10 [14] and IL-12p40 (this study), whether these induced cytokines can fatherly promote IL-32 expression. Quantitative real-time PCR showed that PPE32 stimulation resulted in an increase in IL-32αβ (Figure 3A), IL-32γ (Figure 3B) and IL-32δ (Figure 3C) isoforms after 12 h incubation. Similarly, the same results were repeated in lung endothelial cells A549 co-incubated with PPE32 protein (Figure S2).

### PPE32 promotes cytokines production via ERK1/2 signaling pathway activation

**Figure 4:**
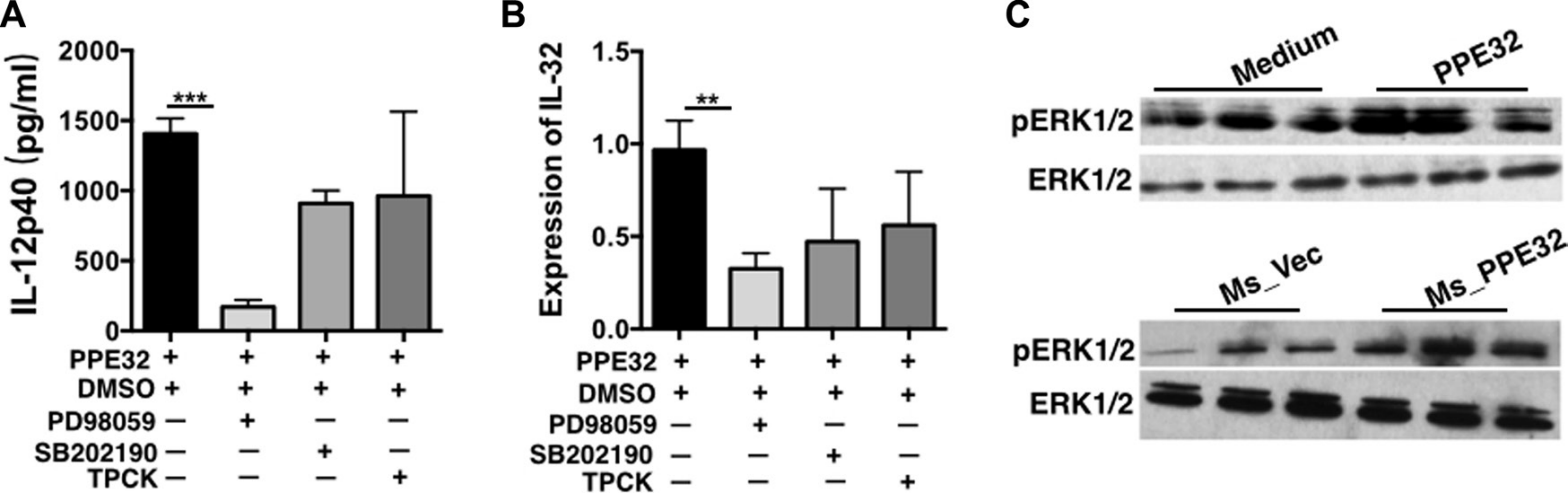
Signaling pathways underlying PPE32-stimulated pro-inflammatory response in THP-1 macrophages Macrophages were treated with specific inhibitors of ERK (PD98059), p38MAPK (SB202190) and NF-κB (TPCK) for 1 h before incubation with PPE32 (5 μg/ml). (**A**) Macrophage was treated with PPE32 for indicated time points; ELISA was used to detect the secretion of IL12p40. (**B**) The expression of IL-32 was determined by quantitative RT-PCR. (**C**) Macrophages were infected with Ms_Vec and Ms_PPE32, or incubated with PPE32 protein for indicated time points, and the phosphorylation of ERK1/2 (pERK1/2) analyzed by Western Blot. In addition, anti-actin antibody was used to confirm that equivalent amounts of the samples were loaded into the gels.

To elucidate the signaling pathway underlying PPE32 promoted cytokine secretion, THP-1 cells were pretreated with specific inhibitors of several cell signal pathways for 1 h and then incubated with PPE32 for 12 h. Cytokines including IL12p40 and IL-32 were quantified by ELISA and RT-PCR. As shown in Figure 4A and 4B, the ERK 1/2 inhibitor (PD98059) significantly decreased the PPE32-induced IL-12p40 and IL-32 production, and the p38MAPK inhibitor (SB202190) and NF-κB inhibitor (TPCK) induced a lower degree of reduction without significant difference in PPE32 treated group and control group. Theses suggest that the PPE32-induced cytokines might primarily depend on the phosphorylation of ERK 1/2. To verify this hypothesis, we performed a Western Blot to measure the phosphorylation of ERK1/2 in THP-1 cells induced by PPE32 stimulation (Figure 4C). The results showed that the phosphorylation of ERK1/2 was significantly enhanced.

### PPE32 suppresses the cell viability of macrophages

**Figure 5:**
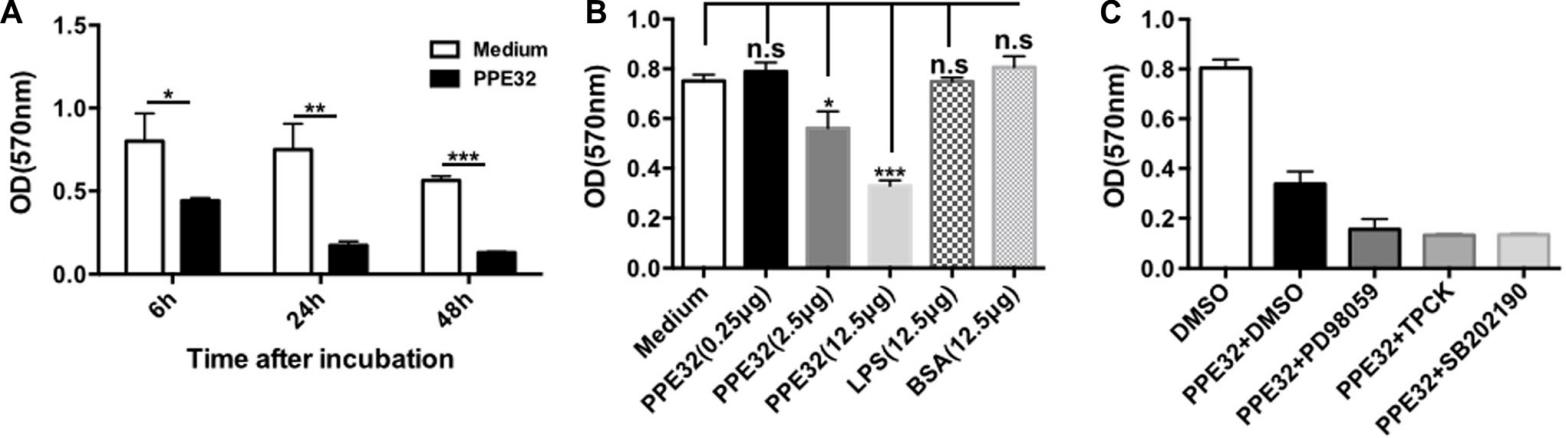
PPE32 suppresses cell viability of macrophages (**A**) Macrophages were incubated with various concentration of PPE32 protein from 0.25 to 12.5 μg/ml for indicated time point and addition of 10 μl 5 mg/ml MTT, after; 3–4 h incubation, solubilization buffer was added and the OD570 was measured. (**B**) Macrophages were incubated with PPE32 for 6 h and concentrations of PPE32 were determined on the basis of cell viability. (**C**) Macrophages were pre-treated with inhibitors targeting ERK1/2(PD98059), p38 MAPK(SB202190), NF-κB(TPCK) 1h before PPE32 stimulation, after 6 h incubation, the cell viability were detected by MTT assay.

To test whether MTB PPE32 can induce cell death in macrophage we tested the effects of PPE32 on cell viability of cells using various concentritions. Macrophages were incubated with PPE32 protein for 6, 24 and 48 h, the cell viability of macrophage were detected by MTT. PPE32 significantly inhibits the cell viability of macrophages after 6 h incubation (Figure 5A). The treatment of PPE32 protein (2.5 μg) efficiently suppresses the cell viability of macrophages in comparison with that of LPS stimulation (Figure 5B). In addition, macrophages cannot resume viability if pre-treated with p38 MAPK, ERK and NF-κB inhibitors (Figure 5C). Lactate dehydrogenase (LDH) release assay was used to discriminate the detailed pathway for PPE32 effect on viability, namely necrosis or apoptosis. LDH assay involves measurement of NADH oxidation to NAD by LDH (released in to the medium from ruptured cells) in the presence of pyruvic acid. THP-1 macrophages were stimulated with different concentrations of PPE32; LDH release was measured in the cultural supernatants. PPE32 has no effect on LDH release of THP-1 macrophages (Figure S3), suggesting that PPE32 might induce apoptotic cell death of macrophage.

### PPE32 induces macrophage apoptosis

**Figure 6:**
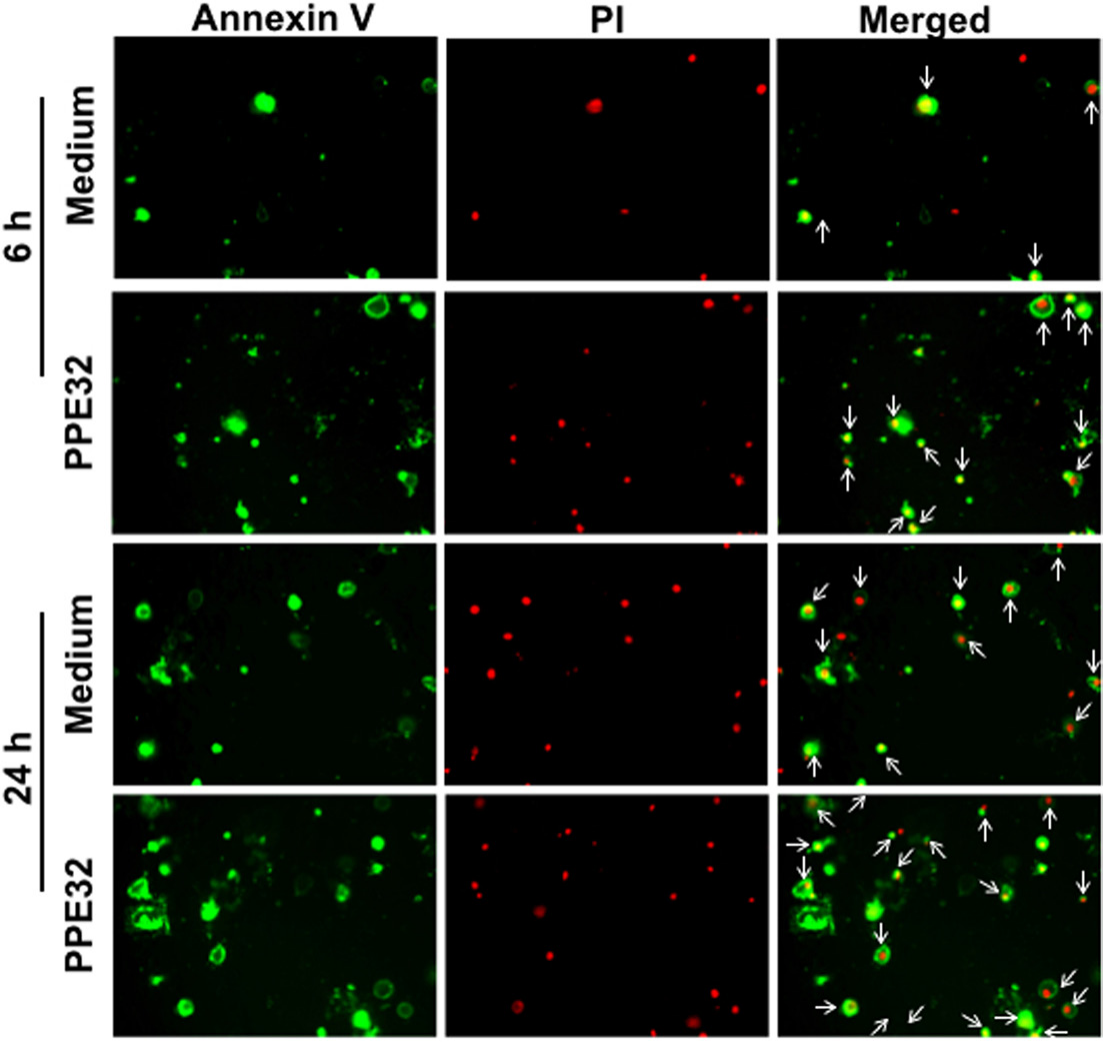
Induction of apoptosis in PPE32 stimulated THP-1 macrophages Macrophages were incubated with 5 μg/ml PPE32 for 6 and 24 h, the cells were washed and subjected to Annexin V/PI strain. The results were visualized by fluorescence microscopy. Early apoptotic populations (Annexin V^+^/PI^−^, green color) or late apoptotic populations (PI^+^/Annexin V^+^, yellow color) and Necrotic (PI^+^/Annexin V^−^, red color) populations were compared.

**Figure 7:**
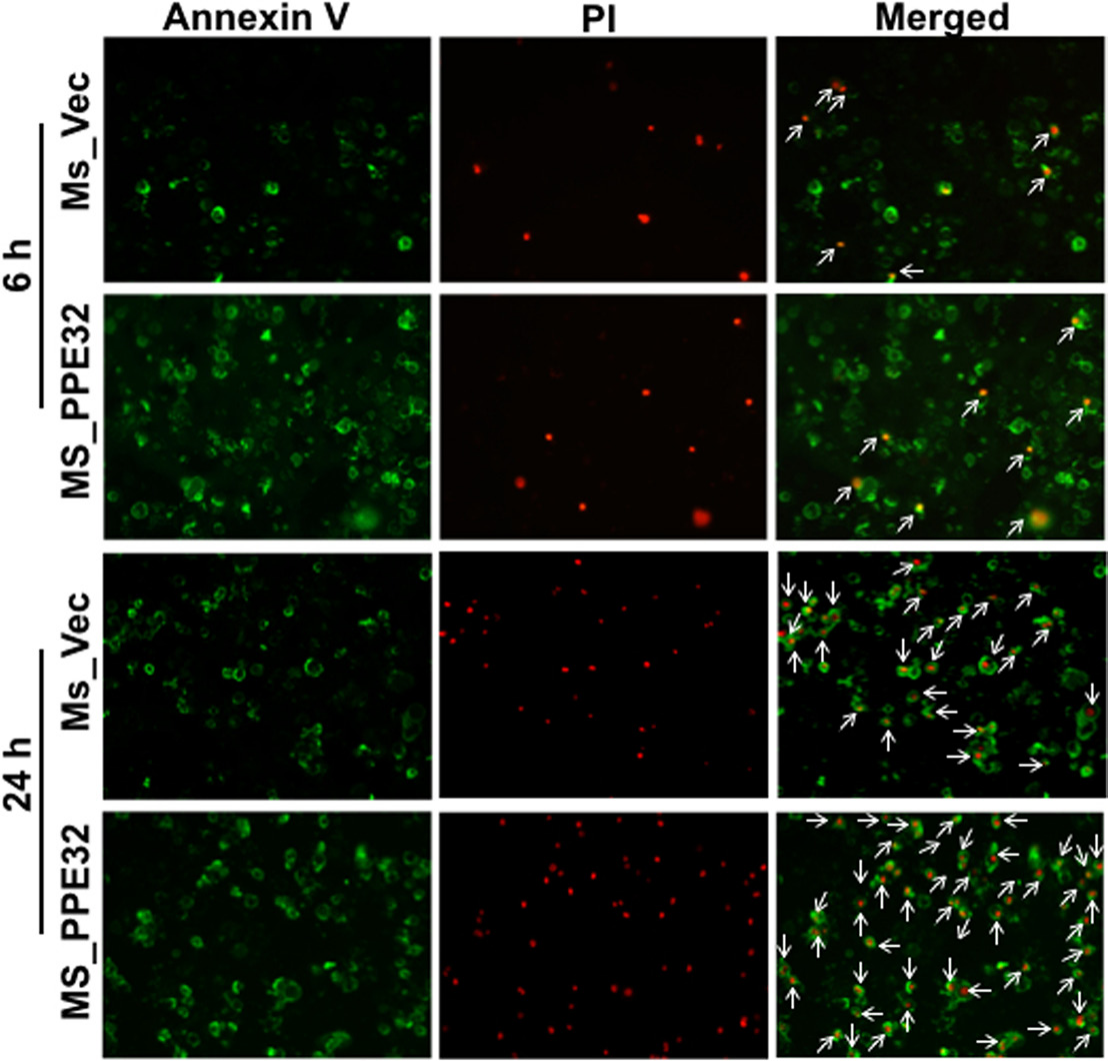
Induction of apoptosis in Ms_PPE32 infected THP-1 macrophages Macrophages cells were infected with MS_Vec and MS_PPE32 at MOI of 10. After 6 and 24 h infection, the cells were washed by cold PBS and subjected to Annexin V/PI strain. Staining with Annexin V/PI was carried out, cells acquired and data analyzed by fluorescence microscope. Early apoptotic populations (Annexin V^+^/PI^−^, green color) or late apoptotic populations (PI^+^/Annexin V^+^, yellow color) and Necrotic (PI^+^/Annexin V^−^, red color) populations were compared between cells infected MS_Vec and MS_PPE32 for 6 and 24 h.

To confirm whether PPE32 can induce cell apoptosis, propidium iodide (PI)/Annexin V staining was performed using macrophages stimulated with PPE32. Propidium iodide easily passes through the ruptured membrane of dead cells and stains nucleic acids, but live cells or cells in early apoptotic phase are impermeable to PI dye. Annexin V binds to phosphatidylserine with high affinity, which is externalized on the surface of apoptotic cells or dead cells, thus recognizing cells undergoing apoptosis even at early stage of cell death. Macrophages stains with the Annexin V/PI detects phosphatidylserine exposure on the outer leaflet of the plasma membrane validated this result. Existence of apoptotic population Annexin V^+^/ PI^−^and Annexin V^+^/ PI^+^ double positive cells suggest that PPE32 protein induces the apoptosis of macrophages in the early and late stage of cell death (Figure 6), as validated from pulmonary epithelial cells A549 stimulated by PPE32 for 6 h (Figure S4). In addition, MS_PPE32 infected macrophage showed more Annexin V^+^/ PI^−^ and Annexin V^+^/ PI^+^ cells than MS_Vec infected macrophage (Figure 7). These results suggest that PPE32 has the ability to induce cell apotopsis in macrophages.

### PPE32 enhances the expression of macrophages caspase-1

**Figure 8:**
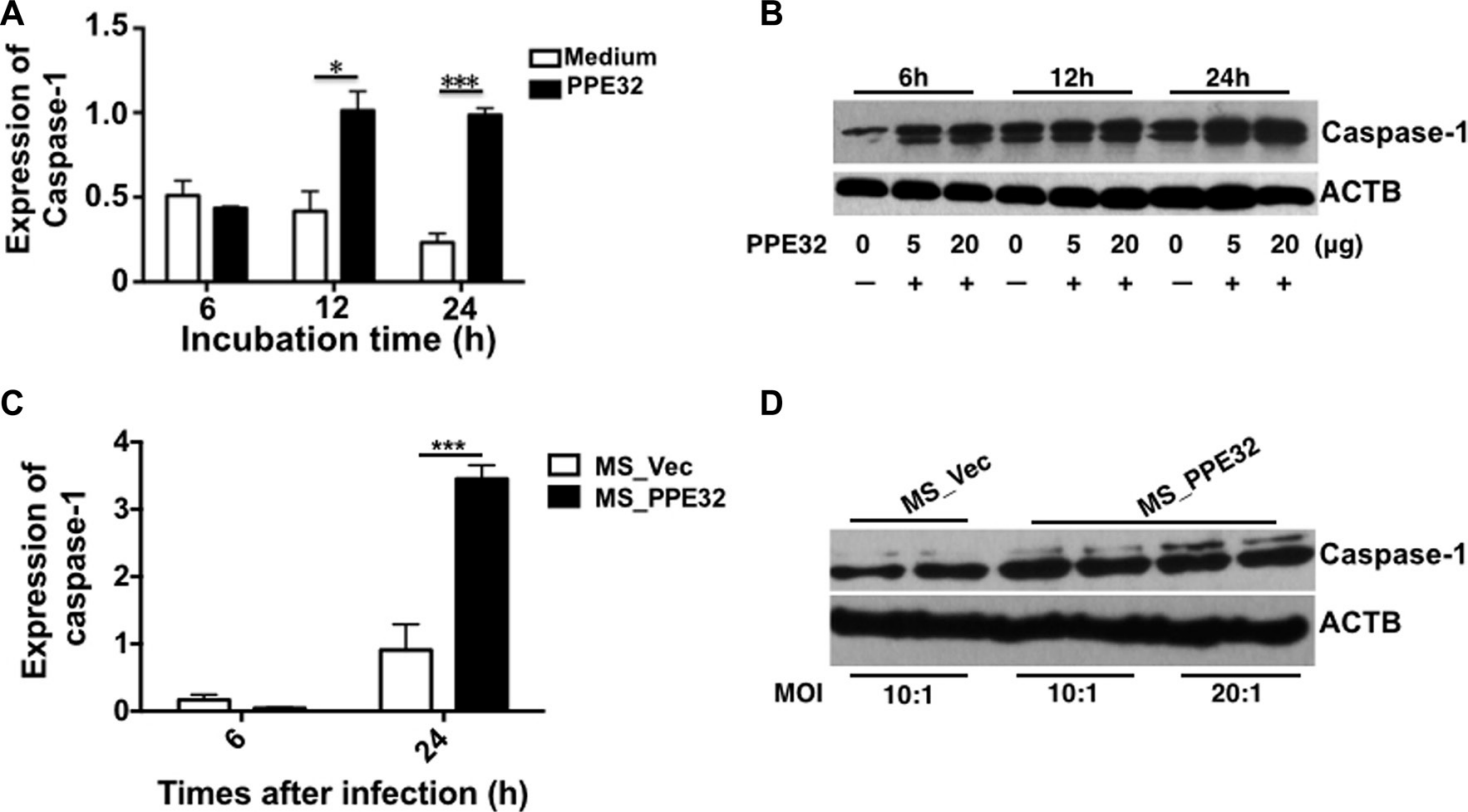
PPE32 promotes the expression of caspase-1 (**A**) Macrophages were incubated with 5 μg/ml PPE32, after 6, 12 and 24 h incubation; the transcription of caspase-1 was detected by RT-PCR. (**B**) Macrophages were incubated with the different concentration (5 μg/ml and 20 μg/ml) of PPE32 protein for 6,12 and 24 h. Cold PBS washed the cells and the cell lyses were subjected to Western Blot. (**C**) Macrophages were infected with MS_Vec and MS_PPE32 at MOI of 10 and 20 for 6 and 24 h, the transcription of caspase-1 was detected by RT-PCR. D. Macrophages were infected with MS_Vec and MS_PPE32 for 24 h. Cold PBS washed the cells and the cell lyses were subjected to Western Blot. ACTB serves as an internal control.

Caspase-1 is critical regulators in innate immunity and several important inflammatory diseases [44]. Caspase-1 activation is involved in inflammation and the regulation of immune responses and differentiation [45]. IL-32 and TSLP production are also increased by the activation of caspase-1 [45, 46]. We are interested in the correlation between PPE32-induced IL-32 and caspase-1. To this end, macrophages were exposed to PPE32, or infected with MS_Vec, or MS_PPE32. PPE32 activates caspase-1 after 12 and 24 h stimulation (Figure 8A) and the expression of caspase-1 were also induced after 6, 12 and 24 h treatment (Figure 8B). In addition, caspase-1 mRNA (Figure 8C) and expression of caspase-1 (Figure 8D) were also enhanced in the macrophages after infection with Ms_PPE32 in comparison with MS_Vec. The same result was noted in the PPE32 treated A549 cells (Figure S5). These results suggest that PPE32 has the ability to induce expression of caspase-1, a key player involved in IL-32 activated pyroptosis of macrophages.

### PPE32 induces the cell apoptosis is caspase dependent

**Figure 9:**
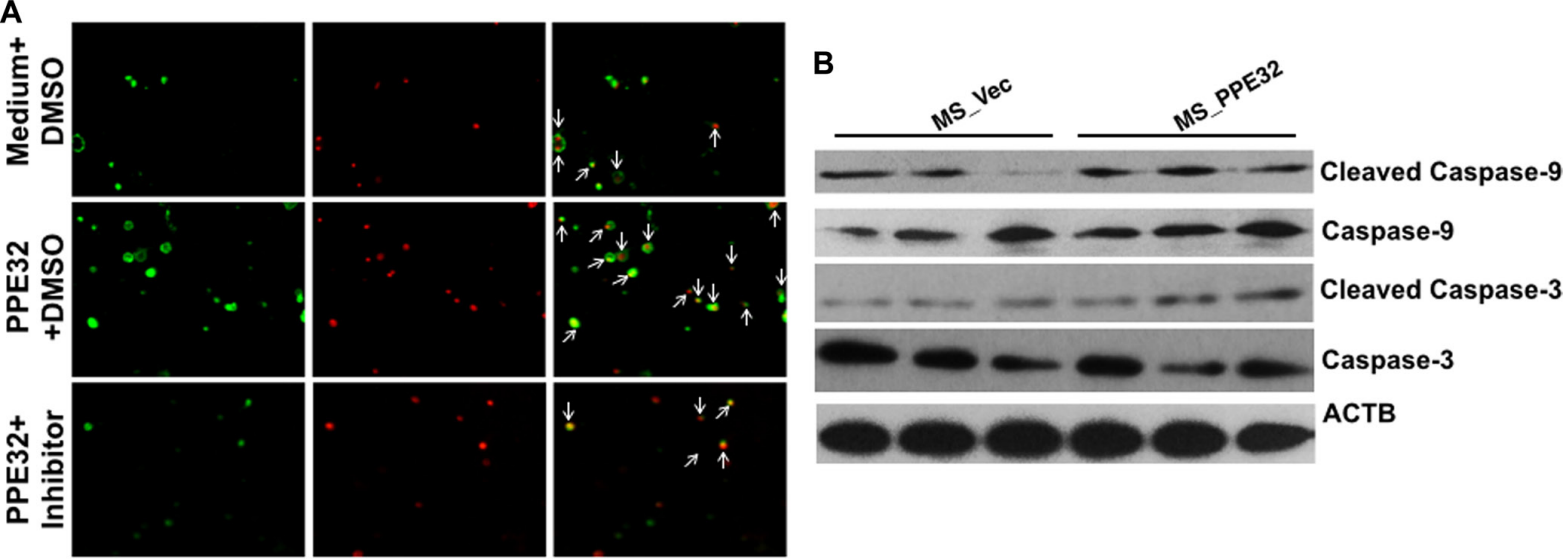
PPE32 activates cell apoptosis via caspase cascade (**A**) Caspase inhibitor Z-VAD-FMK (20 μm) pre-treated macrophage for 1 h, cells were incubated with 5 μg/ml PPE32 for 6 h and subjected to Annexin V/PI strain and the data analyzed by fluorescence microscopy. (**B**) THP-1 cells were infected with MS_Vec and MS_PPE32 at MOI of 10 for 24 h, the whole cell lysates were subjected to Western blot to detect the cleaved caspase-3 and caspase-9.

Apoptosis is a defense mechanism that initiates both innate and adaptive immunity [47]. The caspase-1 was induced in both THP-1 macrophages and A549 cells upon incubation with PPE32. We speculated that caspase might be engaged in PPE32 induced cell apoptosis. To determine this hypothesis, a specific pharmacologic caspase inhibitor (Z-VAD-FMK) was used. THP-1 cells were treated with Z-VAD-FMK (20 μM) for one hour before PPE32 stimulation. After 6 h incubation, the cells were stained with Annexin V/PI. Compared to control cells, PPE32 significantly increases Annexin V/PI positive cells, while partial abrogation of PPE32-activated cell apoptosis by treatment with caspase inhibitor Z-VAD-FMK (Figure 9A). In addition, PPE32 activates the executioner caspase-3 and caspase-9 cleavage by Western blot analysis using specific antibodies against the cleaved caspase 3 and caspase-9 (Figure 9B).

### ER stress associated genes are induced by PPE32

**Figure 10:**
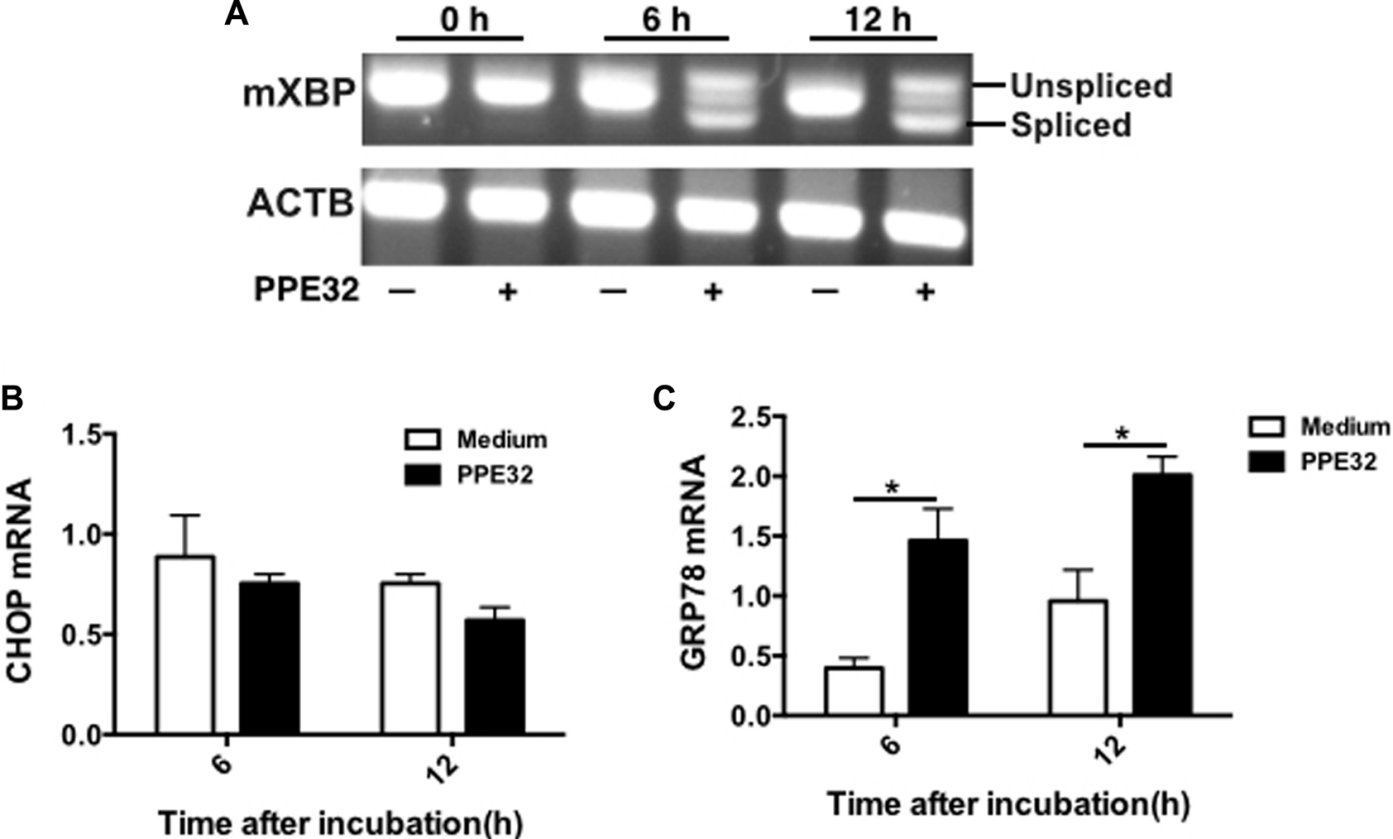
ER stress is involved in PPE32-inducedt macrophage apoptosis (**A**) Macrophages were stimulated with 5 μg/ml PPE32 for 6 and 12 h. Total RNA was isolated and 1.0 μg of total RNA was used to generate cDNAs by reverse transcription PCR and amplified using XBP-1 specific primers that were used to amplify products of unspliced and spliced mRNA. After RT-PCR, the amplified products were separated by electrophoresis on a 3% agarose gel and visualized by ethidium bromide staining. (**B**) The expression of CHOP mRNA in macrophages after incubation with PPE32. (**C**) The expression of GRP78 mRNA in macrophages after treatment with PPE32. The statistical significance of observed differences between antigen stimulated and unstimulated groups were verified by student *t*-test.

The endoplasmic reticulum (ER) stress response is a cellular mechanism that aids in protecting the ER against ER stresses and is involved in ER stressor-induced apoptosis [48]. In order to investigate the relationship between PPE32, the ER stress response and cell apoptosis, we examined X-box binding protein-1 (XBP-1) splicing of macrophage after PPE32 incubation. It is well known that a transcription factor involved in the ER stress response, is induced by ATF6 and spliced by IRE1. As shown in Figure 10A, the spliced XBP-1 mRNA was markedly increased at 6 h after stimulation with PPE32 in macrophages (Figure 10A). To test the activation of major chaperone proteins by mycobacterial PPE32, we analyzed the 78-kDa glucose-regulated protein (GRP78) and C/EBP homologous protein (CHOP) expression, both known to be associated with ER stress [49]. The expression of GRP78 mRNA was increased after 6 and 12 h treatment by PPE32 treatment (Figure 10B), while no difference was detected in CHOP mRNA expression after PPE32 treatment (Figure 10C).

## DISCUSSION

The success of MTB within hostile intracellular niche largely contributes to its host manipulation [50]. Understanding the complex interaction between the MTB and the host, especially how the pathogen entry dormant non-replicating status and causing latent TB infection [51], can inform better control measures. PE/PPE, which are characterized by highly conserved N-terminal domains and diverse roles in MTB pathogenesis [6, 52]. Although the pathophysiological and immunological role of MTB PE/PPE proteins have yet to be comprehensively understood, recent evidence has shown PE/PPE proteins serve as effectors for immune evasion during MTB infection [19, 53].

Cytokines important for host defense against MTB and other pathogenic mycobacteria include TNF-α [54, 55], IFN-γ [56], IL-8, IL-12 [57], IL-18 [58], and IL-23 [59]. We previously showed that PPE32 promotes cytokines production, resulting in enhanced intracellular survival of recombinant MS expression PPE32 within the macrophages [14]. In this study, we found PPE32 increase IL-12p40 expression after 6 h stimulation (Figure 2A–2C), while the expression of IL-32 was induced 12 h after PPE32 stimulation (Figure 3). Studies have shown that IL-32 has pro-inflammatory properties in that it can induce production of TNF-α, IL-6, MIP-2, IL-12 and IL-8 [42, 46, 60, 61]. In return, cytokines such as IL- 1β, IFN-γ, IL-18 and IL-12 can induce IL-32 production [62]. These data suggest that PPE32 may promote IL-12p40 production, and fatherly active the expression of IL-32. In addition, PPE32-induced IL-12p40 mRNA (Figure 4A) and IL-32 mRNA (Figure 4B) expression was inhibited when using ERK1/2 inhibitors PD98059, while no difference were detected using p38MAPK and NF-κB inhibitor treatment. Compare to MS_Vec infection, ERK1/2 phosphorylation was enhanced after MS_PPE32 infection, and similar result was found in PPE32 stimulated macrophages (Figure 4C). These data suggest that PPE32 increases the expression of IL-12p40 and IL-32 via ERK1/2 signaling pathway.

Apoptosis of resting macrophages is likely to be a part of the immune defense mechanism. Mycobacteria-induced apoptosis of infected macrophages releases mycobacterial antigen-containing apoptotic vesicles, which engulfed by dendritic cells, and further processing the antigenic cargo for subsequent presentation to CD8 T cells [63]. Compare to control group, Annexin V^+^/PI^+^ cells in macrophage were significantly increased after incubation with PPE32 or infected with Ms_PPE32 (Figures 6 and 7), suggesting that PPE32 has the ability to induce cell apoptosis of macrophage. Several lines of evidence suggest that IL-32 is critical for cell apoptosis associated with MTB infection [41, 64]. We have identified PPE32 promotes IL-32 isoforms expression in macrophage (Figures 3 and S4) and induces cell apoptosis, suggesting PPE32-induced IL-32 expression might be responsible for PPE32-enhanced cell apoptosis. Studies have shown that IL-32 induced cell apoptosis can be both caspase-3 dependent and caspase-3-independent, such as caspase-1-dependent pyroptosis [64]. We found increased caspase-1 expression in PPE32-incubated or MS_PPE32-infected macrophages in comparison with control group (Figure 8), and the cleaved caspase-9 and caspase-3 were enhanced after MS_PPE32 infection (Figure 9B).

Although several proteins of the PE/PPE family have been demonstrated to function individually in cell apoptosis, the underlying mechanism of action remains unknown [24–26]. The endoplasmic reticulum (ER), a network of intracellular membrane sacs extending from the nuclear membrane throughout the cytoplasm, has been found to be associated with MTB protein ESAT-6-mediated apoptosis [65]. Hence, ER stress might underlie MTB PE/PPE family antigen induced apoptosis. We demonstrated that XBP-1 splicing is increased by mycobacterial PE/PPE family antigen PPE32 (Figure 10A). GRP78 is normally associated with ER sensors localized within the ER membrane [66]. The higher level of GRP78 mRNA upon MS_PPE32 infection than by MS_Vec infection suggests the involvement of ER stress (Figure 10C). The activation of caspase in response to ER stress has been suggested as a putative molecular marker of apoptosis [67]. We found increased caspase-9 and caspase-3 cleavage in MS_PPE32 infected macrophages in infection comparison with control group (Figure 9B).

Taken together, our data demonstrated that MTB PPE32 serves as a novel pro-inflammatory protein that activates IL-12p40 and IL-32 expression in THP-1 macrophages via activating the ERK1/2 signaling. Fatherly, PPE32-activated cell apoptosis is caspase dependent and the macrophages showed increased ER stress response after PPE32 stimulation. Overall the above results point to an interesting interplay of signaling modules that are employed by the PPE32 to increase pro-inflammatory response so as to promote cell apoptosis.

## MATERIALS AND METHODS

### Reagents and antibodies

Phorbol myristate acetate (PMA) was obtained from Sigma–Aldrich (Sigma), dissolved in DMSO and stored at −20°C. The final concentration of dimethylsulphoxide (DMSO) in all experiments was less than 0.1%. The ERK inhibitor SB202190, NF-κB inhibitor, N-p-Tosyl-L-phenylalanine chloromethyl ketone (TPCK) and p38 inhibitor SB203580 were purchased from Sigma–Aldrich. Caspase inhibitor Z-VAD-FMK was purchased from Selleck. Cell culture reagents and medium were obtained from Corning/Costar and BD-Falcon. The antibody against total ERK1/2, phospho-ERK1/2 (Thr202/Tyr204), Caspase-1, Caspase-3, Caspase-9 (Human Specific) and ACTB were obtained from Cell Signaling Technology.

### Preparation of mycobacterial proteins and recombinant strains

The PPE32 protein was expressed and purified as described earlier [14]. Briefly, The PPE32 encoding gene was amplified from of MTB H37Rv genome. The PCR product was directly cloned into the pET28 vector (Invitrogen) in frame with a six N-terminal histidine tag for expression and purification. The concentration of protein was determined using BCA Protein Assay Kit (TIANGEN, China) and was incubated with 10% v/v polymyxin B-agarose (Sigma-Aldrich, USA) for 1 h at 4°C [68]. *Mycobacterium smegmatis* mc^2^155 (MS) was used for construction the recombinant strains. PPE32 gene was cloned into pNITmyc vector in frame with a six N-terminal myc-tag (Invitrogen). MS harbored pNITmyc (MS_Vec) and pNITmyc-PPE32 (MS_PPE32) were constructed previously and used in our previous study [14]. These recombinant strains were cultured in Middlebrook 7H9 liquid medium supplemented with 25 μg/ml kanamycin (Kan).

### Analysis *in vitro* response to stress

Recombinant MS strains were grown into optimal concentration (OD600 = 0.8) in 7H9 medium containing 25 μg/ml Kan. Ms_Vec and Ms_PPE32 were performed in presence of stress condition after 16 h induction by ε-caprolactam. MS_Vec and MS_PPE32 were treated by 0.03% SDS for 1, 2, 3, and 4 h. In addition, pH gradient was generated by adding HCl into 7H9 medium, and sterilized. After the treatment, the recombinant strains were ten-fold dilution spotted onto Middlebrook 7H10 agar containing Kan and bacteria numbers were counted after 3 days culture.

### Cell culture and infection

THP-1 macrophages and A549 human alveolar epithelial cells (ATCC CCL-185) were grown in Park Memorial Institute's medium and Dulbecco's modified Eagle's medium (Gibco-BRL, Grand Island, NY) supplemented with 10% FBS and penicillin (100 IU/ml)/ streptomycin (100 μg/ml). PMA-differentiated macrophages were incubated with PPE32 (5 μg/ml) or infected with recombinant Ms_Vec and Ms_PPE32 strains at MOI (Multiplicity of Infection) of 10 for 6 h and 24 h. The culture supernatants were collected from the infected macrophages and subjected to centrifugation at the speed of 3000 × g. Then move the supernatants carefully into another clean tube and keep it into −80°C for using. The cytokines in the culture supernatants were detected using commercially specific ELISA kits.

### Quantitative real-time PCR

The RNA was extracted from the infected cells using RNA extraction kit (Roche) and mRNA level of cytokines were detected by quantitative RT-PCR using gene specific PCR primers listed in Table 1. Probe Master Mix kit (Roche Applied Science) and miScript SYBR Green PCR kit (Qiagen) were used for amplification of IL12p40, IL-32, CHOP and GRP-78 or internal control β-actin (ACTB).

**Table 1:** The primers used in this study

Primer	Sequence (5′-3′)
IL12p40 (F)	GGACCAGAGCAGTGAGGTCTT
IL12p40 (R)	CTCCTTGTTGTCCCCTCTGA
IL32αβ (F)	CTGAAGGCCCGAATGCACCAG
IL32αβ (R)	GCAAAGGTGGTGTCAGTATC
IL32γ (F)	GTAATGCTCCTCCCTACTTC
IL32γ (R)	GCAAAGGTGGTGTCAGTATC
IL32δ (F)	TCTCTGGTGACATGAAGAAGCT
IL32δ (R)	GCAAAGGTGGTGTCAGTATC
Caspase-1 (F)	GAAGGTACAATAAATGGCTTAC
Caspase-1 (R)	GAATAACGGAGTCAATCAAA
XBP-1 (F)	AAACAGAGTAGCAGCTCAGACTGC
XBP-1 (R)	TCCTTCTGGGTAGACCTCTGGGAG
GRP78 (F)	GTATTGAAACTGTAGGAGGTGTC
GRP78 (R)	TATTACAGCACTAGCAGATCAG
CHOP (F)	GCACCTCCCAGAGCCCTCACTCTCC
CHOP (R)	GTCTACTCCAAGCCTTCCCCCTGCG
**β-actin (F)**	TTCCTTCCTGGGCATGGAGTCC
**β-actin (R)**	TGGCGTACAGGTCTTTGCGG

### Measurement of XBP-1 splicing

XBP-1 encodes a potent transcriptional activator and serves as a substrate for IRE1 RNase. IRE1 RNase cleaves XBP-1 mRNA and the spliced XBP-1 mRNA induces the expression of ER chaperones and other genes involved in the degradation of misfolded proteins [69]. To amplify XBP-1 mRNA, PCR was performed for 30 cycles (94°C, 30 s; 55°C, 30 s; 72°C, 1min), Spliced and unspliced fragments of XBP-1 (454 bp and 480 bp) were separated by 3% agarose gel electrophoresis [65]. β-actin is being used as an internal control. The primers of XBP-1 and β-actin (ACTB) listed in Table 1.

### Cell viability assays

The cytotoxic effect of PPE32 on macrophages was evaluated using the MTT assay. Briefly, macrophages (1 × 10^5^ cells/well) were seeded into a 96-well culture plate. After adherence and differentiation, cells were treated with various concentrations of PPE32, ERK inhibitor SB202190, NF-κB inhibitor TPCK and p38 inhibitor SB203580, and incubated at 37°C for different time points. Then the cells were incubated with 100 μl (0.5 mg/ml) 3-(4,5-Dimethyl-2-Thiazolyl)-2,5-diphenyl tetrazolium bromide (MTT, Sigma Aldrich) for 4 h at 37°C. The formazan crystals were solubilized with DMSO, and the absorbance was measured at 570 nm with a microplate reader (Molecular Devices, USA). After 6 h incubation, medium was removed from each well. For data evaluation, background and reference wavelength corrected absorption values were averaged for the triplicates and expressed as “Cell viability (%)” referring to the untreated control containing only the solvent DMSO.

### Cell apoptosis assays

The apoptosis of macrophages in the presence of PPE32 was determined using the Annexin V/propidium iodide (PI) apoptosis kit (Sigma–Aldrich) according to the manufacturer's instructions. Briefly, the 1 × 10^5^ macrophages and A549 were exposed to PPE32 at final concentration of 5 μg/ml or MS_Vec and MS_PPE32 for 6 and 24 h. After washing with cold PBS, cells stained with 10 μl FITC-conjugated Annexin V antibody (green) and 5 μl PI (red) in 400 μl of Annexin binding buffer (10 mM HEPES pH 7.4, 2.5 mM CaCl_2_ and 140 mM NaCl). The mixture was incubated for 10 min at room temperature in dark and the results were analyzed by multichannel fluorescence microscope.

### Western blot

PPE32 was incubated with the macrophages for different intervals. The macrophages were washed with cold PBS and lysed in an ice-cold RIPA buffer containing protease inhibitors. The cell lysates were centrifuged at 12,000 g for 10 min at 4°C. Then, the supernatants were collected and quantified using the BCA method (TIANGEN Biotechnology, China). The whole cell lysates were subjected to SDS–PAGE, subsequently blotted onto a NC membrane. The membrane was incubated with the specific antibodies. ACTB was used as an internal control. Rabbit anti-ERK1/2, anti-pERK1/2, Anti-caspase-3, anti-caspase-9 and ACTB antibodies were purchased from Cell Signaling Technology.

### Statistical analysis

All experiments were performed in three times. The differences between groups were analyzed by Graphpad Prism 6 software. The data are given as means ± standard error of the mean (SEM). *P*-value (Statistical significance) was decided using Student's *t*-test. *P*-values were less than 0.05 were supposed to be statistically significant. ^*^P < 0.05, ^**^P < 0.01, and ^***^P < 0.001.

## SUPPLEMENTARY MATERIAL FIGURES


