# Supplementary material for: Mycobacterium tuberculosis PPE32 promotes cytokines production and host cell apoptosis through caspase cascade accompanying with enhanced ER stress response
Source: Oncotarget. 2016 Sep 15;7(41):67347–59. doi: 10.18632/oncotarget.12030 (PMC5341880; doi:10.18632/oncotarget.12030)
Supplement: Supplementary file 1 [file oncotarget-07-67347-s001.pdf]

Mycobacterium tuberculosis PPE32 promotes cytokines production and host cell apoptosis through caspase cascade accompanying with enhanced ER stress response

Supplementary Materials

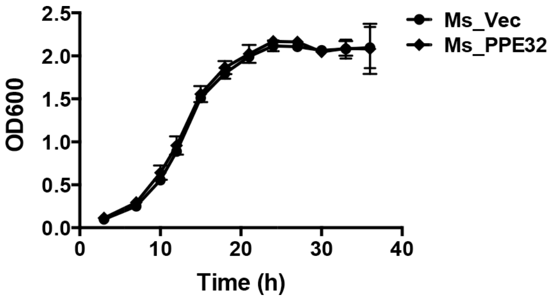

Supplementary Figure S1: *In vitro* growth of Ms_Vec and Ms_PPE32. Ms_Vec and Ms_PPE32 were grown in Middlebrook 7H9 medium supplemented with 0.2% glycerinum, 0.05% Tween 80, 25 μg/ml Kanamycin and 28 mM ε-caprolactam. The OD600 were determined at an interval of 3 h.

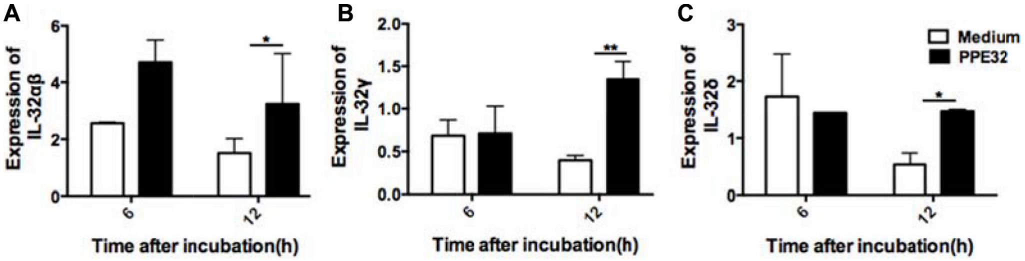

Supplementary Figure S2: PPE32 induces the expression of IL-32 isoforms in A549 cells. A549 cells were incubated with 5 μg/ml PPE32 for 6 and 12 h, levels of IL-32 isoform IL-32αβ mRNA (**A**), IL-32γ mRNA (**B**) and IL-32δ mRNA (**C**) were detected by RT-PCR.

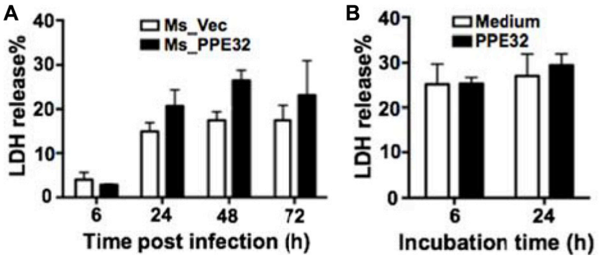

Supplementary Figure S3: PPE32 has no effect on the necrosis of macrophages. Culture supernatants were collected from macrophage infected with Ms_Vec or Ms_PPE32 at MOI of 10 (**A**) or incubated with 5 μg/ml PPE32 protein (**B**). The release of LDH as a measure of macrophage cell death was estimated at various time points. Data are shown as means ± SEM of triplicate wells. Similar results were obtained in three independent experiments.

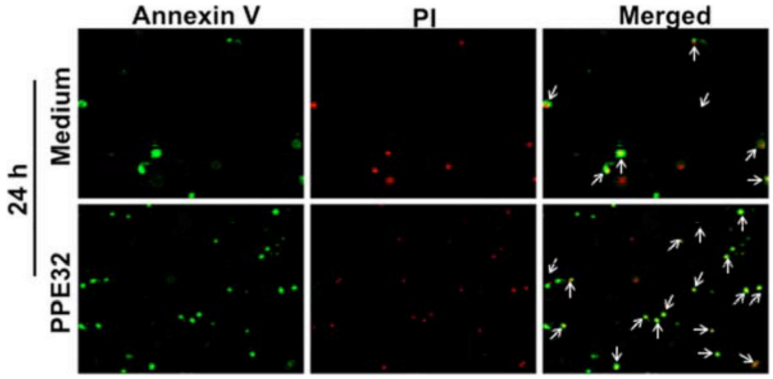

Supplementary Figure S4: Induction of apoptosis in PPE32 stimulated A549 cells. A549 cells were incubated with 5 μg/ml PPE32 with different concentration for 24 h, A549 cells were stained by Annexin V/PI and detected the positive cells by fluorescence microscopy.

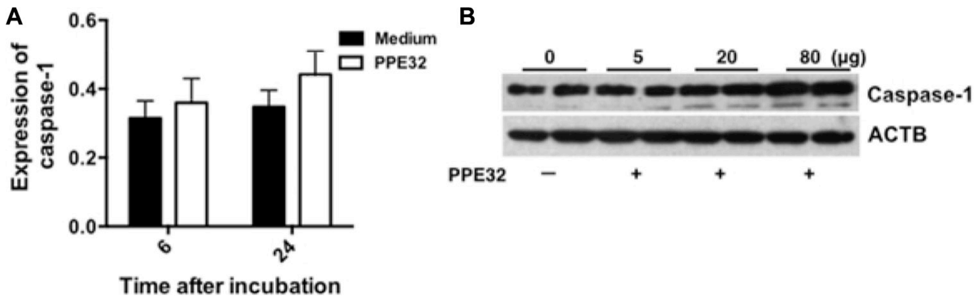

Supplementary Figure S5: PPE32 promotes the expression of caspase-1 in A549 cells. (**A**) A549 cells were incubated with 5 μg/ml PPE32, after 6 and 24 h incubation, the transcription of caspase-1 were detected by RT-PCR. (**B**) Macrophages were incubated with the different concentration (5, 20, 80 μg/ml) of PPE32 protein for 24 h. The whole cell lyses were subjected to Western Blot to detect the expression of caspase-1.
